# Compromised microvascular oxygen delivery increases brain tissue vulnerability with age

**DOI:** 10.1038/s41598-018-26543-w

**Published:** 2018-05-29

**Authors:** Mohammad Moeini, Xuecong Lu, Pramod K. Avti, Rafat Damseh, Samuel Bélanger, Frédéric Picard, David Boas, Ashok Kakkar, Frédéric Lesage

**Affiliations:** 1 Biomedical Engineering Institute, École Polytechnique de Montréal, Montréal, QC Canada; 2 Research Center of Montreal Heart Institute, Montréal, QC Canada; 3 Department of Chemistry, McGill University, Montréal, QC Canada; 4 Centre de Recherche de l’Institut Universitaire de Cardiologie et Pneumologie de Québec (IUCPQ), Québec, QC Canada; 5 Athinoula A. Martinos Center for Biomedical Imaging, Massachusetts General Hospital, Harvard Medical School, Charlestown, MA USA; 6 Biomedical Engineering Department, College of Engineering, Boston University, Boston, MA USA; 7 Present Address: Department of Biophysics, Postgraduate Institute of Medical Education and Research, Chandigarh, India

## Abstract

Despite the possible role of impaired cerebral tissue oxygenation in age-related cognition decline, much is still unknown about the changes in brain tissue pO_2_ with age. Using a detailed investigation of the age-related changes in cerebral tissue oxygenation in the barrel cortex of healthy, awake aged mice, we demonstrate decreased arteriolar and tissue pO_2_ with age. These changes are exacerbated after middle-age. We further uncovered evidence of the presence of hypoxic micro-pockets in the cortex of awake old mice. Our data suggests that from young to middle-age, a well-regulated capillary oxygen supply maintains the oxygen availability in cerebral tissue, despite decreased tissue pO_2_ next to arterioles. After middle-age, due to decreased hematocrit, reduced capillary density and higher capillary transit time heterogeneity, the capillary network fails to compensate for larger decreases in arterial pO_2_. The substantial decrease in brain tissue pO_2_, and the presence of hypoxic micro-pockets after middle-age are of significant importance, as these factors may be related to cognitive decline in elderly people.

## Introduction

It has been established that cognitive function declines even in healthy aging^1–4^. While the exact mechanisms are not known, declined cerebrovascular function has been the subject of intense investigation due to its crucial role in oxygen supply to the neuronal units^5^. Clinical findings show a correlation between cognitive impairment and vascular disorders^6–8^ as well as between cognitive impairment and cerebral blood flow (CBF)^3,9^. These correlations suggest that gradual changes in the brain microvasculature and oxygen delivery which occur with aging may significantly contribute to cognition decline.

Cellular studies suggest that restricted oxygen supply can contribute to neuronal death and increased incidence of cognitive impairment by promoting reactive oxygen species (ROS) formation and calcium dyshomeostasis^10,11^. Strikingly, it has been shown that hypoxia, even *in vitro*, promotes the formation of amyloid β peptide, which is believed to be the primary neurotoxic element of Alzheimer’s disease (AD)^10,11^. There are several lines of evidence supporting the possible role of disrupted cerebral oxygenation in cognition decline. Clinical studies suggest that conditions which lead to restricted oxygen delivery to the brain promote the onset of cognitive disorders. In the extreme case of a stroke, the likelihood of developing dementia is several-fold higher in subjects that survive the event^10,11^. Oxygen supply to the brain is also globally decreased during high altitude excursions^12^ which are associated with cognitive defects in both human^13–15^ and experimental animals^16^. Furthermore, it has been shown in rats that intermittent hypoxia leads to increased incidence of neuronal death in the cortex^17^.

Despite its critical importance, to our knowledge, there is no data available in the literature regarding brain tissue pO_2_ (oxygen partial pressure) changes with age. Previous aging studies investigating brain oxygenation were limited to near-infrared spectroscopy (NIRS) and functional magnetic resonance imaging (fMRI) measurements of attenuated oxygenated and total hemoglobin concentration response to hypercapnic challenges^18^ or neuronal activation^19–23^. Translation of these findings to tissue pO_2_ is not straightforward because cerebral tissue pO_2_ is affected by several other factors, including CBF, geometry and morphology of the vascular network, capillary density, and the cerebral metabolic rate of oxygen consumption (CMRO_2_); all of which are potentially modulated by age. Furthermore, the brain tissue pO_2_ distribution is highly heterogeneous^24^ leaving open the possibility of microscopic hypoxic domains as opposed to homogeneous pO_2_ decrease.

Here we study age-related changes in brain tissue oxygenation within the barrel cortex of healthy, awake aged mice. In addition, vascular pO_2_, capillary flow and non-capillary blood flow parameters were measured to investigate the underlying vascular substrates for observed tissue oxygenation changes.

## Results

### Aging is associated with lower oxygenation of cerebral arterioles and venules

**Figure 1:**
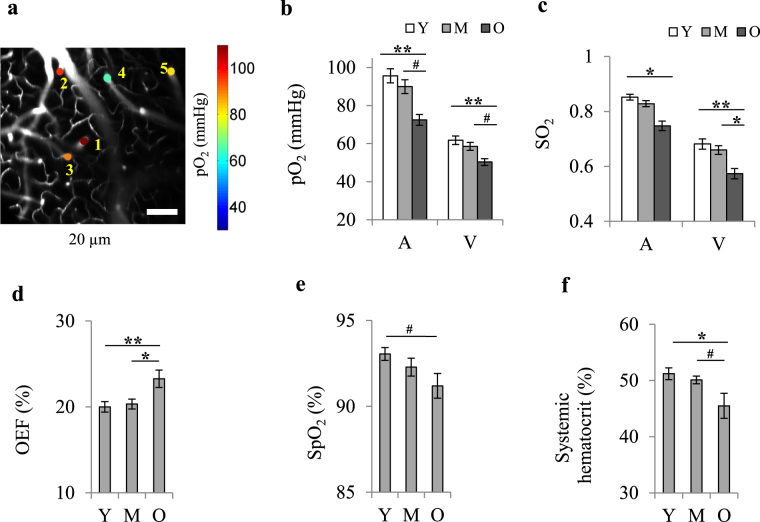
Decreased cerebral vascular oxygenation with age. (**a**) Examples of vascular pO_2_ imaging in diving arterioles (1–2) and ascending venules (3–5) at the depth of ~20 µm in a young mouse. The scale bar is 100 µm. (**b**,**c**) Changes in mean arteriolar (A) and venular (V) pO_2_ and SO_2_ with age. Mean pO_2_ and SO_2_ for each vessel were obtained by averaging the values over the first 150 µm of depth. pO_2_ values were converted to SO_2_ using the Hill’s equation (for arterioles: Y, n = 21; M, n = 21; O, n = 26 vessels; for venules: Y, n = 23, M, n = 25, O, n = 31 vessels; data from 8 young, 8 middle-aged, and 8 old mice). (**d**) Oxygen extraction fraction (OEF) versus age, obtained from SO_2_ values in (**c**). Error bars were obtained from SO_2_ standard deviations using the theory of error propagation. (**e**) Arterial oxygen saturation (SpO_2_) measured by pulse oximetry on the tail (Y, n = 15, M, n = 14, O, n = 14 mice). (**f**) Systemic hematocrit measured on a separate batch of mice (age-matched) by centrifugation of blood samples (Y, n = 10, M, n = 9, O, n = 8 mice). Y: young, M: middle-aged, O: old. Bar plots represent mean ± s.e.m. Statistical significance was calculated using ANOVA followed by Tukey HSD post hoc test. **p < 0.01 *p < 0.05, ^#^p-value approaches significance (p < 0.1).

We first performed direct measurements of vascular pO_2_ in penetrating arterioles and venules of young (6–8 month-old), middle-aged (13–15 month-old) and old (24–26 month-old) mice (n = 8 in each) using two-photon phosphorescence lifetime microscopy^24^ (Fig. 1a, Supplementary Fig. 1a) and intravascular injection of the O_2_-sensitive two-photon enhanced phosphorescent dye PtP-C343^25^. All measurements were performed in awake mice on a treadmill wheel (Supplementary Fig. 1b) to avoid possible age-related anesthesia confounds. Cerebral imaging was performed through thinned-skull cranial window preparations^26^. We observed reduced oxygenation levels in both arterioles and venules with age, which was aggravated and reached statistical significance after middle-age (Fig. 1b,c). Average pO_2_ in diving arterioles was 94.7 ± 4.0, 86.9 ± 3.9 and 75.0 ± 4.0 mmHg in young, middle-aged and old mice, respectively. Average pO_2_ in ascending venules was 62.9 ± 3.2 (young), 58.1 ± 2.7 (middle-aged) and 49.9 ± 2.1 (old) mmHg. Oxygen extraction fraction (OEF) was higher in the old mice (Fig. 1d), which correlates with measurements acquired in human studies performed with MRI and PET^27–29^.

Arterial oxygen saturation (SpO_2_) measured via the tail using pulse oximetry also showed a decreasing trend (Fig. 1e). This conveys a global decrease in arterial oxygenation with age, which was magnified in smaller arterioles of cerebral tissue potentially due to a high oxygen consumption rate. Age-related decline in arterial pO_2_ has also been reported in human studies^30–32^. Lower arterial oxygenation in older mice correlated with a decrease in systemic hematocrit (Fig. 1f), although disturbed alveolar capillary gas exchange in older subjects due to altered respiratory mechanics, pulmonary ventilation/perfusion mismatch and increased alveolar dead space has also been suggested to play a role^30,32^.

### Brain tissue pO_2_ decreases with age and becomes spatially more heterogeneous

**Figure 2:**
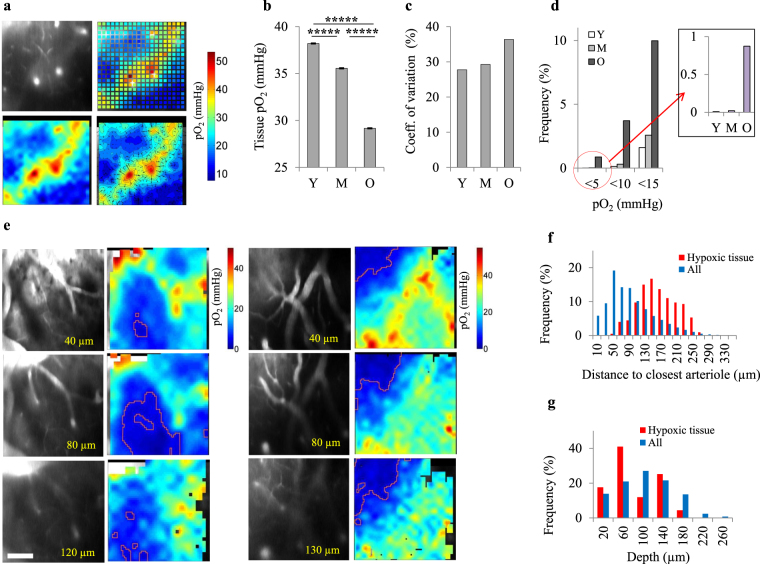
Compromised cerebral tissue oxygenation in old ages. (**a**) A representative pO_2_ grid over a 400 µm × 400 µm region at the depth of 130 µm; top-left: fluorescent image showing the vasculature, top-right: pO_2_ grid measurements, bottom-left: interpolation of 2D grid image, bottom-right: contour plots of bottom-left image. (**b**) Average tissue pO_2_ decreased with age (Y, n = 27037 sampled points from 7 mice; M, n = 23549 sampled points from 6 mice; O, n = 25974 sampled points from 7 mice). The bars represent mean ± s.e.m. Statistical significance was calculated using ANOVA followed by Tukey HSD post hoc test. *****p < 0.00001. (**c**) Spatial heterogeneity of pO_2_ distribution in tissue (defined as coefficient of variation = SD/mean) increased with age. (**d**) The percentage of sampled points with low pO_2_ increased with age. In young (Y) and middle-aged (M) mice, percentage of hypoxic points (pO_2_ < 5 mmHg) is negligible, but in old (O) animals, about 1% of sampled points are hypoxic (inset figure). (**e**) Two examples of hypoxic micro-pockets (marked with red lines) observed in old mice. We found hypoxic tissue in 3 old mice (out of 7 imaged old mice). The scale bar is 100 µm. (**f**) Distribution of the distance of sampled points to closest arteriole for all points and hypoxic points (pO_2_ < 5 mmHg) in old group, showing a negative correlation between the chance of hypoxia and distance to closest arteriole. (**g**) Distribution of the depth of sampled points for all points and hypoxic points (pO_2_ < 5 mmHg) in old group, showing no clear correlation between the chance of hypoxia and depth. Y: young, M: middle-aged, O: old.

The 3D distribution of pO_2_ in cerebral tissue was imaged in a separate batch of mice (7 young (8–9 month-old), 6 middle-aged (15–16 month-old) and 7 old (26–28 month-old)) up to a depth of 250 µm (Fig. 2a). For tissue measurements, the PtP-C343 dye was slowly injected into the brain tissue, rather than by intravascular injection.

Average tissue pO_2_ decreased with age, with a more substantial decline after middle-age (Fig. 2b). Average tissue pO_2_ was 38.2 ± 0.1, 35.6 ± 0.1 and 29.2 ± 0.1 mmHg in young, middle-aged and old mice, respectively. The decrease in average tissue pO_2_ correlated with arteriolar pO_2_ (Fig. 1b,c) with a similar trend. However, changes in the capillary network and microvascular blood flow also played a role in the altered microscopic distribution of oxygen in tissue with aging (see below). Lower average tissue pO_2_ was also associated with increased spatial heterogeneity of O_2_ distribution in the old mice (Fig. 2c), which could originate from capillary network dysfunction making the aged brain susceptible to tissue hypoxia in micro-domains.

### Evidence for the presence of hypoxic micro-pockets in the aged cortex

We then investigated if old animals with a lower and more heterogeneous cerebral tissue pO_2_ develop hypoxic regions. Reported values in the literature for critical tissue pO_2_ range from 0.01–9 mmHg, with the majority of values between 1–5 mmHg^33^. Thus, when seeking pockets of hypoxia, we used pO_2_ < 5 mmHg as a strong indicator that tissue is at, or approaching, pathologically low oxygenation. Although the exact pO_2_ value which induces neuronal injury is not well defined, this arbitrary definition indicates a potential risk of such injury. Investigation of the data revealed that old mice had a higher frequency of sampled points with low pO_2_ (Fig. 2d) with almost 1% of the points that were hypoxic (Fig. 2d, inset). On the other hand, the number of hypoxic sampled points was negligible in the young and middle-aged mice. We then investigated the spatial organization of these hypoxic points. In old mice, low pO_2_ points were co-localized in the form of hypoxic micro-pockets with a size reaching up to ~200 µm in some regions (Fig. 2e). No hypoxic micro-pockets were detected in young or middle-aged mice. Analysis of the distances of these hypoxic points to the closest arterioles and their depth showed that they occur more often far from arterioles (Fig. 2f), in the capillary bed, but there was no correlation with depth (Fig. 2g).

### Changes in non-capillary blood flow with aging

**Figure 3:**
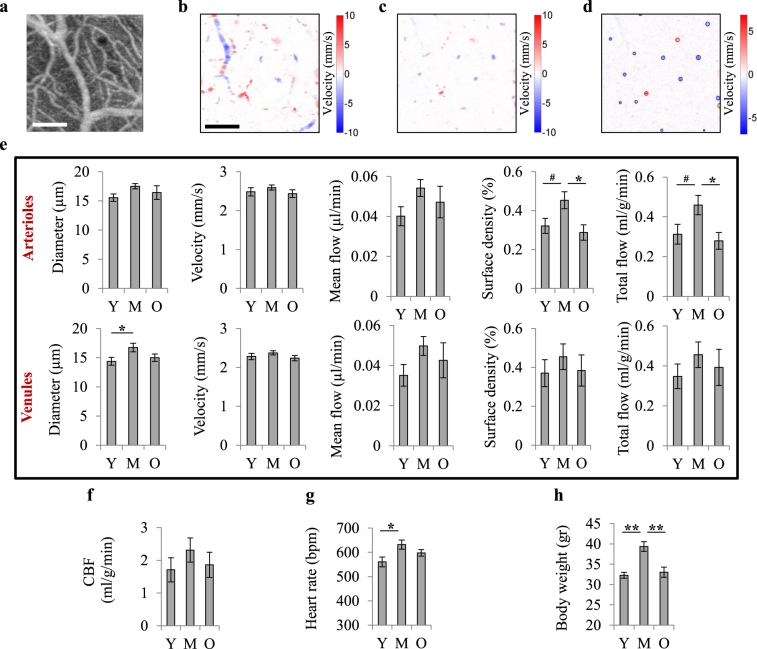
Non-capillary blood flow. (**a**) *En face* maximum intensity projection (MIP) of a 3D OCT angiogram through the depth of 0–650 µm. The scale bar is 200 µm. (**b**) *En face* MIP of a 3D OCT velocity volume through the depth of 0–650 µm, on the same region as in (a). Positive velocity represents downward flow (arterioles) and negative velocity represents upward flow (venules). The shifts in flow direction in surface vessels are due to their curved path. (**c**) *En face* MIP of a 3D OCT velocity volume through the depth of 50–100 µm, on the same region as in (**a**). The top 50 µm volumes were excluded to remove the surface vessels. Only 50 µm of depth just below the surface vessels was included for the estimation of total blood flowing into or out of brain in the imaged region. (**d**) An *en face* slice through the OCT velocity volume at a cortical depth of approximately 70 μm, over the same region as in (**a**). For each slice in the OCT velocity volume, arterioles and venules were detected (red and blue circles) and their diameter, average velocity and flow were obtained. The summation of flow and cross-section area of individual vessels over the slice yielded the total arterial or venular flow and the surface densities of vessels over the region at each depth. (**e**) Diameter, velocity and flow of individual vessels detected in the slices of the OCT velocity volume, as well as total arterial and venular flow and surface densities were averaged through the depth of 50–650 µm to yield the mean values over the imaged region in each animal. Top 50 μm cortical layer was excluded to remove surface vessels (top row: arterioles; bottom row: venules). (**f**) For each animal, arterial and venular total flows, averaged through the depth of 50–100 µm, were averaged as an estimate of regional CBF. (**g**) Heart rate, extracted from pulse oximetry data. (**h**) Body weight. Y: young (n = 14), M: middle-aged (n = 14), O: old (n = 15). Bar plots represent mean ± s.e.m. Statistical significance was calculated using ANOVA followed by Tukey HSD post hoc test. **p < 0.01, *p < 0.05, ^#^p-value approaches significance (p < 0.1).

Blood flow in non-capillary vessels (diameter > 10 µm) was studied using Doppler Optical Coherence Tomography (OCT)^34^ (Supplementary Fig. 1f and Fig. 3) in mice from both vascular and tissue pO_2_ experiments (n = 14, 14 and 15 for young, middle-aged and old groups, respectively). As expected, the total blood flow values obtained from penetrating arterioles and venules were almost equal (Fig. 3e, column 5). An inverted U-shaped trend, increase from young to middle-age and decrease thereafter, was observed in all flow parameters (Fig. 3e) although the trend was very weak for blood velocity and flow in individual vessels and did not reach statistical significance in all cases. Most importantly, we observed a clear inverted U-shaped trend in total blood flow which reached statistical significance for arterioles. This inverted U-shaped trend in total flow originated mainly from the higher surface area of the vessels at middle-age (Fig. 3e, column 4), which resulted from increased vascular diameter (Fig. 3e, column 1), and not the number of vessels (Supplementary Fig. 2).

CBF estimates from average arteriolar and venular flow maps were 1.7 ± 0.4, 2.3 ± 0.4 and 1.9 ± 0.4 ml/g/min for young, middle-aged and old mice, respectively (Fig. 3f). Estimated CBF followed an inverted U-shaped trend with age which did not reach statistical significance. Three previous studies using the same method reported a CBF of ~0.6 ml/g/min in anesthetized young rats^34^ and 0.5–2.0 ml/g/min in anesthetized young mice under varying conditions^35,36^. One study using the quantitative autoradiographic iodo[14 C]antipyrine method obtained a CBF value of ~0.7 ml/g/min for the barrel field of anesthetized young rats, but CBF was ~1.8 ml/g/min under awake conditions^37^, which is in agreement with our CBF estimate in the barrel cortex of young mice.

Interestingly, heart rate (HR, extracted from pulse oximetry data) and body weight of the animals also followed the same inverted U-shaped trend (Fig. 3g,h). Data from Barsha *et al*. also shows that in awake, non-restrained mice, HR follows an inverted U-shaped trend from 3 to18 months of age, peaking at 14 months^38^. A number of studies on mice^39^, monkeys^40^ and humans^41^ have shown that cardiac output (CO) is directly linked to HR. Therefore, the observed increase in HR at middle-age suggests increased CO, which is necessary to support a higher demand for oxygen and other nutrients in middle-aged mice having a larger body mass. Indeed, a study found lower CO in 2-month-old rats compared with 6- and 24-month-old rats, but CO normalized to total body mass was not different among age groups^42^. We also observed that HR normalized to the body weight did not differ in our age groups (Supplementary Fig. 3a). A higher CO at middle-age correlated with the higher total observable brain arterial flow.

### Capillary network remodeling from young to middle-age maintains capillary bed tissue pO_2_ despite lower tissue pO_2_ adjacent to arterioles

**Figure 4:**
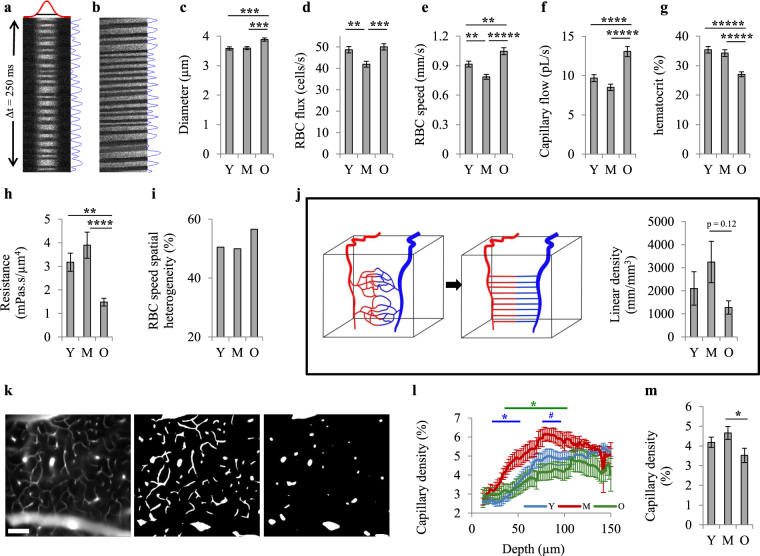
Capillary blood flow. (**a**) A representative space-time image from perpendicular scans. The image was averaged in vertical direction and fitted with a Gaussian function (top) to estimate the capillary diameter. The image was also averaged in horizontal direction (right) to find the number of passing RBCs. (**b**) A representative space-time image from longitudinal scans. RBC velocity was calculated from the angle of dark streaks. The image was rotated by this angle and averaged (right) to find the number of passing RBCs. (**c**–**g**) Capillary diameter (**c**), RBC flux (**d**) and RBC speed (**e**) were obtained from space-time images. Capillary volumetric flow (**f**) and hematocrit (**g**) were calculated from diameter, flux and speed (Y, n = 242; M, n = 252; O, n = 278 capillaries). (**h**) Capillary resistance was estimated from diameter and hematocrit. (**i**) Spatial heterogeneity (defined as the coefficient of variation) of RBC speed. (**j**) For the mice that both flow in large vessels and capillary flow were measured over the same region (Y, n = 8; M, n = 7; O, n = 8 mice), capillary linear density was estimated (right) by simplifying the capillary network architecture as straight tubes with uniform length and diameter connecting an arteriole to a venule (left). (**k**) Microvascular angiograms were used to directly obtain the capillary density. Left: an *en face* slice at the depth of ~80 µm (scale bar: 100 µm). Middle: binarization of the left image. Right: the same as middle image, but processed with a median filter to remove the fine structures (capillaries). (**l**) Capillary density (volume%) versus depth obtained from angiograms by subtracting the density of large vessels from total vascular density. The blue * (or #) represents statistically significant (or nearly significant) differences between Y and M. The green * represents statistically significant differences between M and O. (**m**) Average capillary density through the depth of 0–150 µm (Y, n = 18; M, n = 15; O, n = 16 angiograms). Results are presented as mean ± s.e.m. Statistical significance was calculated using ANOVA followed by Tukey HSD post hoc test. *****p < 0.00001, ****p < 0.0001, ***p < 0.001, **p < 0.01, *p < 0.05, ^#^p-value approaches significance (p < 0.1). Y: young (8 mice), M: middle-aged (7 mice), O: old (8 mice).

Red blood cell (RBC) flow in capillaries (diameter < 10 µm) was imaged using two-photon laser-scanning fluorescence microscopy (Supplementary Fig. 1a and Fig. 4a,b)^43,44^. Capillary flow measurements (30–40 capillaries per animal) were performed on the mice in which vascular pO_2_ was imaged (n = 8, 7 and 8 for young, middle-aged and old groups, respectively).

From young to middle-age, there was no significant change in capillary diameter, flow, and hematocrit (Fig. 4c,f,g), but RBC flux and speed were reduced (Fig. 4d,e). Since total flow tended to increase from young to middle-age (Fig. 3e), this observation suggests increased capillary density at middle-age such that slightly higher blood flow is distributed into a larger number of capillaries. Using the CBF measure over the same region and assuming a simplified model we evaluated this hypothesis (Fig. 4j). In this model, we assumed that the imaged cortical volume was fed and drained by a single arteriole and venule, and that the capillary network consisted of parallel straight tubes with uniform length and diameter connecting the arteriole to the venule. Mass conservation states that total flow into and out of the imaged volume (CBF) should be equal to the average volumetric capillary flow multiplied by the number of capillaries. Although far from reality, the model provides a rough estimation of the relative capillary densities between the age groups. Estimated capillary densities (Fig. 4j) showed an increasing trend from young to middle-age, which did not reach statistical significance. We then calculated the capillary density from two-photon fluorescent angiograms (Fig. 4k). Capillary density at middle-age was higher when compared with the young age at most depths in the range 0–150 µm, which reached statistical significance (p < 0.05) through the depths of ~25–55 µm and approached significance through the depths of ~75–90 µm (Fig. 4l).

**Figure 5:**
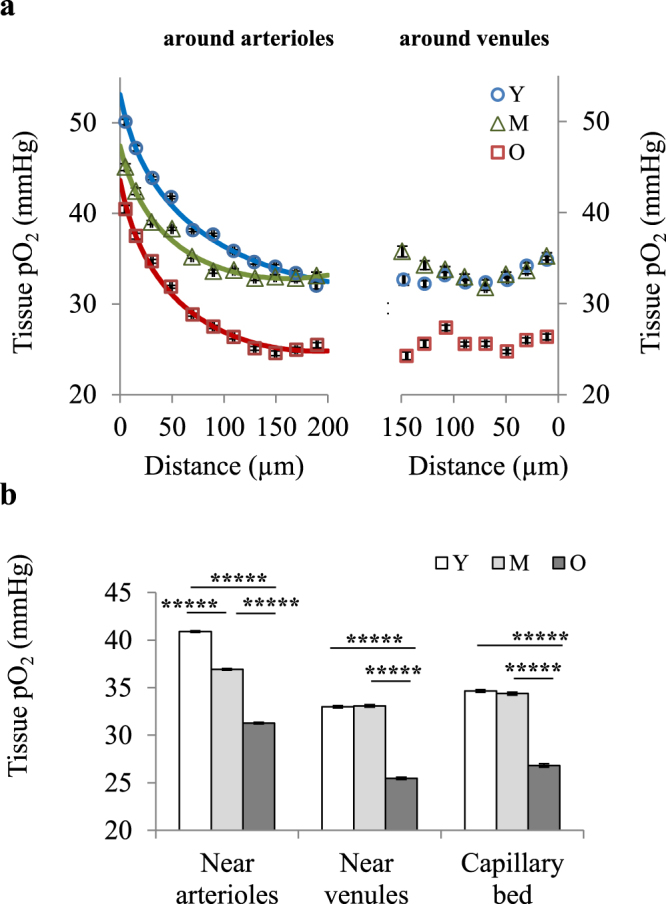
Region-specific tissue oxygenation changes with age. (**a**) For each sampled pO_2_ point, the distance to closest arteriole and venule was obtained. All sampled points in each age group were then pooled and plotted versus distance from closest arteriole or venule to obtain average tissue pO_2_ gradients around arterioles (left) and venules (right). Results are presented as mean ± s.e.m. (**b**) Average tissue pO_2_ in three defined regions: near arterioles (<100 µm from an arteriole; Y, n = 17052; M, n = 13971; O, n = 16240 sampled points), near venules (<100 µm from a venule, but >100 µm from closest arteriole; Y, n = 6212; M, n = 6001; O, n = 7834 sampled points), and in capillary bed (>100 µm from arterioles or venules; Y, n = 3773; M, n = 3577; O, n = 1951 sampled points). Results are presented as mean ± s.e.m. Statistical significance was calculated using ANOVA followed by Tukey HSD post hoc test. *****p < 0.00001. Y: young (7 mice), M: middle-aged (6 mice), O: old (7 mice).

Since from young to middle-age, hematocrit did not change (Fig. 4g) and there was no increase in spatial heterogeneity of RBC speed (Fig. 4i) (as an indirect measure of capillary transit time heterogeneity (CTTH) which has been suggested to affect the oxygen extraction efficiency from the capillary network^45,46^) a denser capillary network at middle-age is hypothesized to be able to deliver the same amount of oxygen to the tissue by a smaller pO_2_ gradient (between the capillaries and tissue). This smaller gradient leads to unchanged tissue pO_2_ from young to middle-age despite a decrease in intravascular pO_2_, provided that CMRO_2_ does not change significantly (see below). Interestingly, regional analysis of tissue pO_2_ data confirmed this conclusion. We observed that tissue pO_2_ near arterioles declines continuously with age, but distant from the arterioles (in the capillary bed) tissue pO_2_ is maintained until middle-age followed by a significant decrease afterwards (Fig. 5).

**Figure 6:**
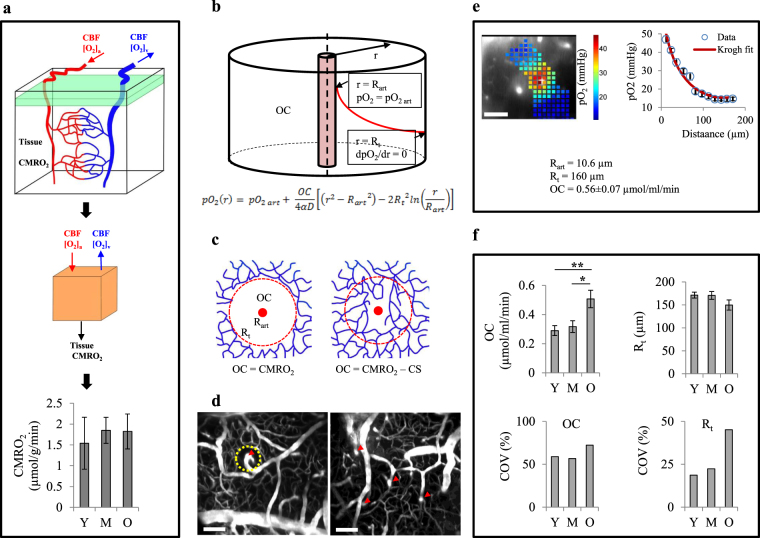
Estimation of cerebral metabolic rate of oxygen consumption (CMRO_2_) and net oxygen consumption rate (OC) in tissue. (**a**) CMRO_2_ was obtained using the equation CMRO_2_ = CBF([O_2_]_a_-[O_2_]_v_) for the mice that both vascular pO_2_ and blood flow in large vessels were measured over the same region (Y, n = 8; M, n = 7; O, n = 7 mice). (**b**) The Krogh cylinder model of O_2_ diffusion from a vessel to surrounding tissue. This model assumes that an infinitely long arteriole with radius R_art_ supplies an infinitely long tissue cylinder with radius R_t_. Continuity equation for O_2_ is solved assuming a uniform net oxygen consumption rate (OC) in tissue cylinder with specified boundary conditions. (**c**) If there is a capillary depleted cylinder around arterioles and the major tissue pO_2_ gradient around arterioles occurs within this region, OC term in Krogh model will be a good estimate of CMRO_2_ (left). But if the capillary depleted region is much smaller than R_t_, OC represents the difference between CMRO_2_ and capillary O_2_ supply (CS) (right). (**d**) Examples of the maximum intensity projection of microvascular angiograms showing diving arterioles (arrow heads) with (left) and without (right) a capillary depleted region; in mice the absence of capillary depleted regions around some arterioles was observed. Even in cases where a capillary depleted region existed, its radius did not exceed 50 µm, while tissue pO_2_ profiles often saw a significant pO_2_ drop up to 100 µm from arterioles. (**e**) 2D-grid point measurements around arterioles (left) yielded pO_2_ gradients from arterioles which were fitted with the Krogh model to obtain OC (right). (**f**) OC and R_t_ were found for individual vessels and were averaged in each age group (top). Spatial heterogeneity of OC and R_t_ were estimated by the coefficient of variation (bottom) (Y, n = 26 arterioles from 5 mice; M, n = 20 arterioles from 5 mice; O, n = 37 arterioles from 7 mice). Y: young, M: middle-aged, O: old. Scale bars are 100 µm. Bar plots represent mean ± s.e.m. Statistical significance was calculated using ANOVA followed by Tukey HSD post hoc test. **p < 0.01 *p < 0.05.

There is conflicting data in the literature regarding CMRO_2_ changes with age; while a few studies found increased CMRO_2_ with age^27,29^, most studies report a decrease or no change in CMRO_2_^28,47–49^. Here, since for each animal we had the measures of arteriolar and venular pO_2_ and also recorded CBF over the same region, it was possible to estimate CMRO_2_ of the imaged volume for individual mice (Fig. 6a). Estimated CMRO_2_ did not change significantly with age (Fig. 6a), supporting our delivery assumptions above.

Next, we investigated the O_2_ delivery to tissue by capillaries to assess our hypothesis of unaltered total capillary O_2_ supply from young to middle-age. In rats, it was shown that fitting the tissue pO_2_ profiles from diving arterioles with the Krogh cylinder model of O_2_ diffusion^50^ (Fig. 6b) yields a measure of CMRO_2_^51^. This method was based on the fact that, in rats, there is a capillary depleted region around arterioles^33^ and tissue pO_2_ profiles plateau mainly within this region. Thus, the O_2_ consumption term in the Krogh model is solely representative of CMRO_2_. However, in mice we either do not see capillary depleted regions around arterioles or they are smaller than the distance at which the tissue pO_2_ profiles from arterioles plateau (Fig. 6d). Therefore, Krogh fitting in mice yields a net oxygen consumption rate (OC), which we defined as CMRO_2_ minus the rate of oxygen supply by capillaries (Fig. 6c). Tissue pO_2_ profiles around arterioles, obtained from tissue pO_2_ maps, were fitted with the Krogh model (Fig. 6e) to obtain the OC and R_t_ (the radius of equivalent cylinder supplied by the arteriole). No significant change in OC and R_t_ or their spatial heterogeneities was observed between young and middle-aged mice (Fig. 6f). Since OC and CMRO_2_ did not change significantly from young to middle-age, it supports the conclusion that overall capillary O_2_ supply remains unchanged.

### Impaired capillary function in old mice could be largely responsible for more heterogeneous tissue pO_2_ and the presence of hypoxic micro-pockets

Capillary flow imaging showed that from middle-age to old age there was an increase in RBC flux, RBC speed and capillary flow (Fig. 4d,e,f). Higher RBC flow in old age has been also reported before in anesthetized mice^43^ and rats^44^. Since total flow decreased from middle-age to old age (Fig. 3e), this observation can only be explained by decreased capillary density. Estimated capillary density from CBF and capillary flow data using the simplified parallel capillary tubes model explained above showed a decreasing trend from middle-age to old age, (Fig. 4j). The capillary density calculated from two-photon fluorescent angiograms then provided supporting evidence for decreased density after middle-age (Fig. 4l,m).

We observed an increase in the capillary diameter in the old mice compared with the middle-aged and young animals (Fig. 4a), suggesting a dilation of capillaries to allow a higher capillary flow due to fewer capillaries delivering oxygen. Also, in agreement with lower systemic hematocrit in old mice (Fig. 1f), capillary hematocrit decreased after middle-age (Fig. 4g). The combined effect of decreased hematocrit and increased diameter in old animals reduced the capillary resistance (Fig. 4h) and supported the higher capillary flow.

Finally, we observed increased RBC speed spatial heterogeneity in old mice, which reflects higher CTTH. Thus, the combined effect of higher CTTH, lower hematocrit, lower capillary density and lower vascular pO_2_ levels in the old animals can substantially reduce the capillary O_2_ delivery efficiency and could be largely responsible for the significant decrease in tissue pO_2_ (Fig. 5), increased spatial heterogeneity of tissue oxygenation (Fig. 2c), and the presence of hypoxic micro-pockets (Fig. 2e).

Further evidence for impaired capillary O_2_ delivery in old animals comes from the OC estimations from the Krogh fitting, which was significantly higher in old animals (Fig. 6f). Considering no significant change in CMRO_2_ with age (Fig. 6a), this suggests decreased capillary oxygen supply. In addition, a higher spatial heterogeneity in both OC and R_t_ was observed (Fig. 6f), which suggests a more heterogeneous oxygen supply by capillaries which is in line with our observations of more heterogeneous tissue oxygenation in old animals and the presence of hypoxic micro-pockets.

### Shifted fractional contribution of arterioles and capillaries in O_2_ delivery to cerebral tissue in old ages

Recent evidence showed that both arterioles and capillaries contribute to tissue oxygenation, although capillaries have a higher contribution (~20% by diving arterioles)^52,53^. Our OC and CMRO_2_ values also estimate that ~20–30% of delivered oxygen is supplied by arterioles. As discussed above, CMRO_2_ was not largely affected by age (Fig. 6a). Therefore, in old mice the same amount of total O_2_ should be delivered to the cerebral tissue as in younger animals. Since our data suggests that O_2_ delivery by capillaries is decreased in old mice, arterioles have to provide more oxygen to the surrounding tissue to keep the total O_2_ delivery unchanged.

**Figure 7:**
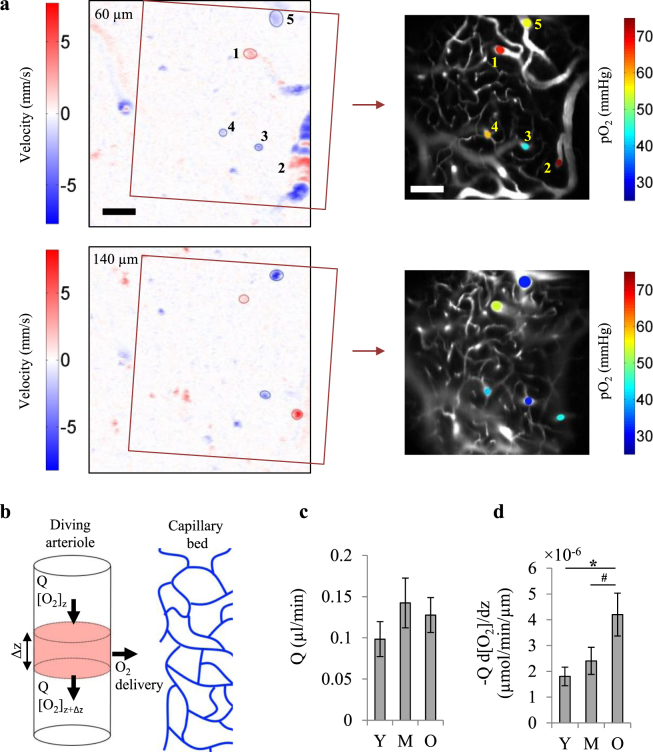
O_2_ delivery by individual arterioles. (**a**) Left: Two *en face* slices through the Doppler OCT velocity volume at cortical depths of approximately 60 and 140 μm. Right: *En face* slices through the two-photon angiogram over specified regions on OCT images (left) at the same depths, with vascular pO_2_ measurements superimposed (1–2: arterioles, 3–5: venules). Vascular pO_2_ was measured in 30–40 µm steps. Measured vessels were identified on the Doppler OCT volume and their average flow over depth was obtained. The scale bars are 100 µm. (**b**) The amount of O_2_ delivered to the tissue by individual arterioles can be estimated from the longitudinal gradient of O_2_ flow in arterioles (O_2_ mass balance). (**c**) Average blood flow of diving arterioles. (**d**) O_2_ delivery to tissue by individual arterioles (per unit length) estimated by longitudinal gradient of O_2_ flow as shown in (**b**). Y: young (n = 14 arterioles from 8 mice), M: middle-aged (n = 13 arterioles from 7 mice), O: old (n = 11 arterioles from 5 mice). Bar plots represent mean ± s.e.m. Statistical significance was calculated using ANOVA followed by Tukey HSD post hoc test. **p < 0.01 *p < 0.05, ^#^p-value approaches significance (p < 0.1).

In the mice that were measured with Doppler OCT and vascular pO_2_ imaging over the same region (n = 8, 7 and 8 mice for young, middle-age and old groups, respectively), we could identify diving arterioles in which we had both the arterial pO_2_ versus depth and their blood flow (Fig. 7a). This technique provided the unique opportunity to calculate the oxygen delivered to the surrounding tissue by individual arterioles (Fig. 7b). The results showed a higher O_2_ delivery by individual arterioles in the old group compared with the young and middle-aged groups (Fig. 7d), confirming our hypothesis of a shift in the fractional contribution of arterioles and capillaries in tissue oxygenation towards arteriolar supply in the old mice. This shift maintains total O_2_ delivery, but brings more heterogeneity to tissue oxygenation. This was also coherent with the observation that hypoxic points were often away from arterioles (Fig. 2f).

## Discussion

In this study, we found age-related decreases in arteriolar and tissue pO_2_ in the mouse cortex, which worsened after middle-age. Our data suggests that from young to middle-age capillary network remodeling maintains the capillary bed tissue pO_2_, despite a decreased tissue pO_2_ next to arterioles. However, after middle-age, oxygen delivery by the capillary network is impaired, with our data pointing to decreased hematocrit, reduced capillary density and higher CTTH as the causes. This results in a significant decrease in tissue pO_2_, higher spatial heterogeneity of tissue oxygenation, and the presence of hypoxic micro-pockets in old mice. Additionally, using a modified Krogh model, we could estimate the capillary oxygen supply and the shift in oxygen delivery from capillaries to arterioles after middle-age, although the larger tissue volumes underlying the CMRO_2_ estimates and the observed variance make a precise comparison difficult to achieve. A limitation of this study was that separate cohorts of animals were used for vascular and tissue pO_2_ measurements. Furthermore, cortical reads do not necessarily represent deep brain regional changes and white matter.

All measurements were done in awake mice, removing the confounding effects of anesthesia. Awake imaging is particularly important in aging studies since anesthesia may affect physiological and hemodynamic parameters differently in young and old subjects. In addition, anesthetized experiments often need mechanical ventilation and/or adjustment of physiological parameters which the results because many of these parameters normally change with aging. To minimize animal stress during imaging, a custom-built treadmill wheel was used in which the animal was able to freely walk or run, while the head was fixed, similar to that of Lyons *et al*.^54^. No obvious difference was observed among age groups in terms of the time it takes for them to habituate to the head restrain conditions or their behavior in the cages, during handling, or on the wheel. In all three age groups, after training sessions, the mice could easily walk on the fixation wheel with no sign of stress or discomfort (e.g., grooming behavior was maintained). While we aimed to minimize confounds, our use of the probe PtP-C343 is also associated with known limitations when measuring absolute pO_2_ values: potential interaction with plasma proteins when doing vascular measures, temperature dependence, O_2_ consumption by the probe itself and background signal that increases with depth, more so in a thin-skull preparation which increases excitation light scattering. However, we have no reason to believe these confounds would differentially affect our group comparisons.

In addition to the role of hematocrit, capillary density and CTTH in decreasing tissue pO_2_ in old mice, a possible shift in the oxyhemoglobin dissociation curve with age can also affect the oxygen uptake in the lungs and its delivery to tissue. In human erythrocytes, it has been shown *in vivo* that RBC aging shifts the oxygen dissociation curve to the left^55–57^. However, it has been reported that the RBC age distribution does not differ in male human subgroups of different ages^56,57^. Investigation of the oxygen dissociation curves of blood samples from different age groups could answer the question more clearly, but we only found one human study showing a slight rightward shift of the oxyhemoglobin dissociation curve in elderly subjects^32^.

Our observation of hypoxic micropockets in old age could be linked to previous neuropathological findings which report that tiny microinfarcts (mean diameter ~200–1000 µm)^58^ are very common in the brain samples from old individuals, particularly people suffering from mild cognitive decline or AD^59^. A causal role of these microinfarcts in age-related cognitive disorders has been suggested. An animal study also reported neuronal loss and cognitive impairment in a mouse model of multiple diffuse microinfarcts^59^. These and our findings support the hypothesis that impaired tissue oxygenation in healthy aging may be one of the mechanisms involved in cognition decline. Unfortunately, in our tissue pO_2_ images the small capillaries were not clearly visible in most cases because of the background fluorescence of the PtP-C343 dye. Therefore, it was not possible to study the correlation between hypoxia and local capillary loss. Although our data showed an overall reduction in mean capillary density and hematocrit in old mice, future studies need to be performed to correlate the hypoxic micropockets with local capillary density or capillary flow parameters, as well as local neuronal death.

The inverted U-shape relationship observed between CBF and age is against the general conception of decreased^60^ or unchanged CBF^61,62^ with age in humans. However, there are a number of studies that report either stable CBF until middle-age with decline thereafter^63–66^, or a parabolic trend in global CBF peaking at an age of ~60 years^67^. In anesthetized rats, CBF was also shown to decrease with age^68,69^, but two studies on conscious rats observed a parabolic trend, peaking between 3–14 months of age^70,71^. Differences in the techniques, brain regions, the age range, the criteria for the selection of healthy old subjects and inclusion or exclusion of middle-aged subjects may account for the disparate findings. Our investigation underlines the necessity of including a middle-age group in aging studies. Our CBF estimates were obtained assuming unchanged cortical thickness with age. As a rough analysis, we normalized the CBF to total body weight and we observed a flatter trend in age-related change (Supplementary Fig. 3b). Although cortical thickness changes do not necessarily follow total body weight changes, this rough analysis showed that observed trends in estimated capillary density and CMRO_2_ were not significantly altered when normalized CBF was used (Supplementary Fig. 3c,d). Investigation of age-related changes in the cortical thickness of mice could provide more accurate CBF assessments with our technique.

In accordance with the CBF data, we also saw a parabolic trend in capillary density. This is in line with the strong correlation between the capillary density and the regional CBF found in rats^72^. Previous studies report decreased, unchanged or increased cerebral capillary density with age^60,73^. Although differences in the techniques, species, brain regions and age range of subjects may explain some of these discrepancies, capillary changes with aging have also been suggested to be biphasic^73^. Indeed, several studies report that capillary density increases until middle-age and then declines during later senescence both in rats^74–76^ and human^77–79^. This indicates a possible capillary response to altered cardiac output or blood pressure, or to meet the metabolic demands. The observed capillary density increase until middle-age in our study could be a regulatory response to decreased vascular oxygen content to maintain the tissue pO_2_ level. However, after middle-age we observed capillary loss possibly due to reduced angiogenesis capabilities. This hypothesis is in agreement with the observations in C57BL/6 J mice showing attenuated angiogenic and neurogenic response to vascular endothelial growth factor (VEGF) stimulation at 24 months of age compared with 3 and 12 months^80^, suggesting maintained capacity for cerebral angiogenesis until middle-age, but a decline thereafter.

Overall, our study reveals an age-related decrease in resting cerebral tissue pO_2_ in conscious mice which was manifested mainly after middle-age. The findings suggest regulated capillary oxygen supply until middle-age to maintain the oxygen availability in the brain tissue. With further aging, tissue pO_2_ decreased significantly, hypothesized to be due to a capillary network failing to compensate larger decreases in arterial pO_2_. The observed substantial decrease in brain tissue pO_2_ and the presence of hypoxic micro-pockets after the middle-age are of significance because they could be involved in neurodegenerative diseases and cognition decline in elderly people.

## Methods

Although this study was not blinded, the analysis of microscopic data was automated and the same parameters and algorithms were used to analyze all images limiting investigator bias. In addition, no data was discarded and care was taken to treat all groups equally during surgery, handling, training and imaging.

### Synthesis of the PtP-C343 probe

The O_2_- sensitive molecule PtP-C343 was synthetized following the general procedures reported in Finikova *et al*.^25^ and Vinogradov *et al*.^81^. Polyethylene glycol (PEG) units with the MW of ~2000Da were used for the PEGylation of the dendrimer periphery. All compounds were characterized using routine techniques, including NMR, mass spectroscopy, GPC, and UV-Vis spectroscopy. The dye was then calibrated at 37 °C and 7.2 pH as described before^82^.

### Animals

Animal handling and surgical procedures were approved by the ethics committee of the research center of the Montreal Heart Institute. All experiments were performed in accordance with the Canadian Council on Animal Care recommendations. Young adult (6–9 month-old), middle-aged (13–16 month-old) and old (24–28 month-old) C57BL/6 J healthy male mice were obtained from the colony of aged mice of the Quebec Network for Aging Research (RQRV) and housed in 12-hr light-dark cycle until imaging. Two batches of mice were used. The mice in batch 1 (7 young (average age = 8.8 ± 0.1 month-old), 6 middle-aged (average age = 15.3 ± 0.1 month-old), 7 old (average age = 27.0 ± 0.1 month-old)) were used to record tissue pO_2_. In batch 2 (8 young (average age = 6.9 ± 0.2 month-old), 8 middle-aged (average age = 14.1 ± 0.1 month-old), 8 old (average age = 24.9 ± 0.1 month-old)), vascular pO_2_ and capillary blood flow were measured. OCT and tail pulse oximetry were performed on both batches. 8–10 days before measurements, a thinned skull window was created over the left barrel cortex under 2.0% isoflurane anesthesia, as described in Shih *et al*.^26^. Briefly, the scalp was removed and a titanium-made head-plate was fixed on the skull using dental cement. The skull was then slowly thinned to translucency with a micro-drill (OmniDrill35, World Precision, USA). A 150 µm-thick cover glass was glued to the window using cyanoacrylate glue and the edges were sealed with dental cement to form a 3 mm diameter cranial window. For tissue pO_2_ measurements, a small thinned region at the edge of the cover glass (~0.5 mm) was left uncovered with dental cement to allow later injection of the PtP-C343 dye into tissue through the soft thinned membrane. During the surgery, animals were fixed on a controlled physiological monitoring system (LabeoTech, Canada) which enabled continuous monitoring of the rectal temperature, respiration and heart rate. Ketoprofen (5 mg/Kg, Merial, Canada) and buprenorphine (0.05 mg/Kg, Reckitt Benckiser Healthcare, UK) were injected before the surgery and baytril (5 mg/Kg, Bayer, Germany) was injected after the surgery. Injections were repeated 24 hours after the surgery.

### Awake imaging

During all measurements, animals were fixed on an angled treadmill wheel which allowed free movement of the limbs while the head was restrained by a titanium bar (Supplementary Fig. 1b). To minimize stress during the imaging sessions, animals were trained on the wheel over four fixation training sessions (starting after 3 days recovery following the surgery) to habituate to the head restraint. The length of time the mice were restrained was gradually increased from 10 to 45 min over 4 sessions.

### Two-photon system

Two-photon imaging was performed using a custom-built laser-scanning microscope (Supplementary Fig. 1a) that used a consecutive sequence of 820 nm, 80 MHz, 150 fs pulses from MaiTai-BB laser oscillator (Newport corporation, USA) through an electro-optic modulator (ConOptics, USA) to adjust the gain and allow the generation of alternating “on” and “off” laser pulse periods for microsecond lifetime imaging. The optical beam was scanned in the x-y plane by galvanometric mirrors (Thorlabs, USA). Reflected light was collected by a 20X objective (Olympus XLUMPLFLN-W, NA = 1). Phosphorescent and fluorescent photons were separated by dichroic mirrors and relayed to two separate photomultiplier tubes (PMTs) for detection of PtP-C343 and dextran-FITC probes. Phosphorescent light was passed through a filter centered at 680 nm and detected by a first PMT (H7422, Hamamatsu Photonics, Japan). Fluorescent light was passed through a filter centered at 520 nm and forwarded to the second PMT (R3896, Hamamatsu Photonics, Japan).

### Capillary blood flow imaging with two-photon fluorescence microscopy

~200 µL 2MDa dextran-FITC (50 mg/ml in saline, Sigma) was injected through the tail vein. Due to the injected fluorescent dye, the plasma appeared bright in the images while RBCs appeared as dark shadows. Capillary flow parameters were obtained from longitudinal and perpendicular line-scans over capillaries using the contrast in the images (see below and Fig. 4). Each line-scan measured 100 points along a straight line (~20 µm long) at a line-rate of 800 Hz. The line-scans were performed continuously and 250 ms segments of the line-scan data (200 lines) were used to create space-time images with dark streaks due to the motion of RBCs (Fig. 4a,b). The space-time images were used to obtain the following parameters for each capillary: (1) diameter by fitting the perpendicular scans with a Gaussian function whose full width at half maximum (FWHM) yielded an estimate of the internal diameter (Fig. 4a); (2) RBC velocity from the angle of the streaks in longitudinal space-time images (Fig. 4b) as described before^44^; (3) capillary volumetric flow (RBC velocity × capillary cross-sectional area); (4) RBC flux (cells/s), determined as the number of dark shadows in the space-time images divided by the acquisition time of the images (the flux was obtained using both longitudinal and perpendicular scans and the average value was used); (5) hematocrit, calculated using the obtained values for RBC flux and volumetric flow^44^ (hematocrit = RBC flux × RBC volume/capillary volumetric flow; RBC volume was considered to be 55 μm^3^ for C57Bl/6 mice^83^); and (6) capillary resistance, estimated from diameter and hematocrit using the equations given in literature^84,85^. All measured capillaries in each age group were pooled for age comparisons. For each animal, 1–3 angiograms (600 µm × 600 µm, 2 µm steps) were also recorded for capillary density calculations.

### Microvasculature segmentation and capillary density calculation

Microvasculature data from two-photon angiograms was segmented using a data-driven approach based on deep learning. Our deep learning model was based on the FC-DenseNet architecture proposed in Jégou *et al*.^86^. The model was composed of 97 convolutional layers with an input size of 256 × 256 × 1. It had 11 dense blocks with 4, 5, 7, 9, 11, 13, 11, 9, 7, 5 and 4 convolutional layers in each block, respectively, with a growth rate of 24. Each block in the first 5 dense blocks was followed by 2 × 2 pooling layers, whereas each block in the last 5 dense blocks was preceded by a 3 × 3 transposed convolution with stride 2 to compensate for the pooling operations. To train our segmentation model, we manually prepared ground truth labels of 8 angiograms captured with our two-photon microscopy. We augmented our dataset by rotation and flipping operations. The training of the model was performed on two NVIDIA TITAN X GPUs using the Theano library in Python. The training was performed by RMSprop optimizer with initial learning rate of 1e−4 and an exponential decay of 0.995 after each epoch. To monitor the training process, we randomly selected 25% of our annotated data as a validation set. We used the validation set to stop the training process based on the mean Intersection over Union (IoU) metric with a patience of 25 epochs. The model was regularized with a weight decay of 1e−5 and a dropout rate of 0.2. We processed the output of the neural network by 3D morphological erosion and dilation operations to refine the segmentations. The vascular density was measured for each slice of the binarized (segmented) two-photon angiograms before and after applying a median filter (Fig. 4k). The application of the median filter removed the fine structures (capillaries). Then, the difference of these two densities (total vascular density and density of large vessels) provided an estimate of the capillary density.

### Tissue pO_2_ imaging with two-photon phosphorescence lifetime microscopy

For tissue pO_2_ imaging, the PtP-C343 dye solution (~150 µM in ACSF) was slowly injected into the brain tissue ~300 µm below the surface with a glass micropipette using a microsyringe injector (UMP3, World Precision, USA). ~200 µl 2MDa FITC-Dextran (50 mg/ml in saline, Sigma) was also injected through the tail vein to visualize the vasculature. For each animal, grid measurements (225–400 points) were performed over 3–4 400 µm × 400 µm adjacent planes at different depths (30–40 µm intervals, up to 250–300 µm deep) (Fig. 2a and Supplementary Fig. 1e). Before each grid measurement, a fluorescent image was recorded to locate the sampling points in the tissue and find their distance to nearby arterioles or venules. At each point, 3000 excitation cycles were averaged before moving to the next point. Each excitation cycle consisted of 25 µs excitation period in which the laser pulse was “on” followed by 275 µs “off” period in which the phosphorescence emission was allowed to decay. Averaged phosphorescence decay at each point was fitted with a single-exponential curve to determine the phosphorescence lifetime. The lifetimes were then converted to pO_2_ using a calibration curve. For all imaging planes, large vessels (diameter > 10 µm) were graphed and labeled as arterioles or venules. The distance of each pO_2_ sampling point to closest arteriole and venule (in 3D) was then found. All sampling points in each age group were pooled to find the average tissue pO_2_, spatial heterogeneity of tissue oxygenation (defined as SD/mean) and the fraction of sampled points with hypoxic pO_2_ (here defined as pO_2_ < 5 mmHg). In addition, pO_2_ profiles from arterioles and venules were obtained by plotting the points versus their distance from closest arteriole or venule. For venular profiles, the points within 100 µm from an arteriole were excluded to avoid the contamination of venular profiles with the dominant effect of arterioles. For a regional study of tissue oxygenation, sampled points were divided into three categories: (1) “near arterioles”, including the points within 100 µm from an arteriole; (2) “near venules”, including the points within 100 µm from a venule, but at least 100 µm from the closest arteriole, and (3) “capillary bed” including the points which were at least 100 µm distant from the closest arteriole or venule. Average tissue pO_2_ was then obtained for each region.

### Estimation of net oxygen consumption rate in cerebral tissue

For simplicity, we assumed a uniform capillary O_2_ supply in tissue, and a modification of the Krogh model^50^ was used to estimate the net oxygen consumption rate in the tissue (OC), defined as CMRO_2_ minus capillary O_2_ supply per unit volume of the tissue (Fig. 6b):

1 $$p{O}_{2}(r)=p{O}_{2art}+\frac{OC}{4\alpha D}[({r}^{2}-{{R}_{art}}^{2})-2{{R}_{t}}^{2}ln(r/{R}_{art})]$$

pO_2 art_ is the arterial pO_2_, R_art_ is the mean arterial radius, R_t_ is the radius of the Krogh cylinder, *α* is oxygen solubility (1.27 × 10^−3^ µmoleO_2_/ml/mmHg)^51^, and *D* is oxygen diffusivity in tissue (~4000 µm^2^/s)^51^. Tissue pO_2_ profiles around arterioles, obtained from tissue pO_2_ maps, were fitted with the Krogh model to find OC and pO_2,art_. R_t_ was determined as the distance at which the curve reached a plateau.

### Vascular pO_2_ imaging with two-photon phosphorescence lifetime microscopy

The PtP-C343 dye solution (~50 mg/ml in saline, ~200 µl) was injected through the tail vein. The fluorescent signal of the dye allowed visualization of the vasculature without the need for FITC-Dextran injection. In each mouse, a few penetrating arterioles and venules were chosen and vascular pO_2_ was measured at 30–40 µm depth intervals, starting right after they branched from pial vessels up to the depth of ~200 µm (Fig. 1a). Each vascular pO_2_ measurement consisted of a line grid measurement with 10–15 points which yielded a parabolic pO_2_ profile whose maximum was used as pO_2_ at the center of the vessel. pO_2_ measurement principles were similar to tissue pO_2_ imaging described above. Obtained pO_2_ values were converted to oxygen saturation of hemoglobin (SO_2_) using the Hill’s equation (for C57BL/6 mice: n = 2.59 and P50 = 40.2)^87^. For each vessel, average pO_2_ and SO_2_ over the first 150 µm of depth were obtained. Average pO_2_ and SO_2_ values were then pooled in each group for age comparisons. Oxygen extraction fraction (OEF = (S_a_O_2_-S_v_O_2_)/S_a_O_2_) was also obtained from average arteriolar and venular SO_2_ values.

### OCT setup

Supplementary Fig. 1f shows a simplified layout of the OCT setup. Light originating from a superluminescent diode (SLD) source (LS2000C, Thorlabs) had a bandwidth of 200 nm, yielding an axial resolution of ~3.5 µm. Light was first sent to a circulator and divided by a 5/95 fiber splitter into reference and sample arms. Scanning on the sample was performed using a dual galvanometer system (Thorlabs) to be imaged using a 5X infinity corrected objective (Edmund Optics, USA) yielding a lateral resolution of 8.5 μm. In each arm, polarization control was integrated to maximize contrast. A custom-built spectrometer based on a volume holographic grating (HD1145, Wasatch Photonics, USA) was used as the detector with a high-speed 2048 pixel line camera (2048 R, Sensors Unlimited, USA) coupled to a 50 mm SWIR lens (Edmund Optics, USA).

### Non-capillary blood flow measurements with Doppler-OCT

For each mouse, an OCT angiogram and a 3D Doppler OCT volume were acquired over a cortical surface of ~700 µm × 700 µm. First, an angiogram volume (512 × 512 pixels) was acquired (Fig. 3a). In each volume, B-scans were repeated twice before moving to the next line. A total of 10 volumes were acquired for averaging purposes at a line rate of 40 Hz. Then, a Doppler OCT scan was performed by oversampling in the x-direction (nx = 2048, ny = 512) to enable the computation of the phase between adjacent overlapping A-lines. The line rate was set to 24 Hz. The Doppler volume was repeated 10 times for averaging. Doppler OCT image reconstruction was done in Matlab. Spectral shaping of the interference signal using a Hanning window was used to eliminate side lobes in the final image at the expense of broadening axial resolution to ~3.8 μm. Automatic dispersion compensation to the second and third order dispersion imbalance was implemented per the procedure described in Wojtkowski *et al*.^88^. Optimization was done on the first frame of each acquisition to obtain the dispersion coefficients which were then applied to the rest of the acquisition. Reconstruction of flow speed was based on a moving-scatterer-sensitive reconstruction technique^34,89,90^ which uses the Kasai Autocorrelator^91^. A digital high pass filter was used to remove the stationary scattering components from the OCT image. For angiography image reconstruction, spectral shaping and dispersion compensation was identical to above. Frame differences were then computed in pairs (for repeated frames) and their variance were averaged over the 10 volumes repetition.

Obtained 3D Doppler OCT datasets consisted of *en face* slices of z-projection blood velocity maps at cortical depth intervals of ~3.8 µm. Pial vessels generated both positive and negative Doppler shifts because of their undulating shape, but penetrating arterioles and venules exhibited pure positive or negative Doppler shifts (Fig. 3b). Therefore, in our analyses we excluded the top 50 µm of the OCT datasets. For each slice in the OCT velocity volume, arterioles and venules were detected by a threshold filter (Fig. 3d) and their minor and major axis lengths, projected area and average projected velocity were measured. Diameter was estimated by minor axis length, flow was calculated as the product of projected area and projected velocity and projected velocity was converted to actual velocity along the vessel path using the ratio of the major and minor axis lengths. Total flow in the imaged region was obtained by summation of the flow in individual vessels over the *en face* plane. This yielded total flow in ml/µm^2^/min, which was converted to ml/g/min by normalizing to the cortical mass corresponding to the scanned area assuming a cortical thickness of 1.5 mm for the barrel field of C57/BL6 mice^92^ and brain density of 1.05 g/ml^93^. All parameters were averaged over the depth of 50–650 µm (Fig. 3e).

It has been established that flow in penetrating arterioles and venules can be assigned to well-defined cortical regions^94^. Thus, total blood flow over the scanning area averaged over the depth of 50–100 µm in which penetrating arterioles and venules have just branched from pial vessels, but before further branching to smaller vessels not detected by Doppler OCT, provided an estimation of regional CBF (Fig. 3f)^34,36^.

### Identification of arterioles, venules and capillaries

The camera images taken during the surgery were used to identify pial arterioles and venules based on their morphology; pial arteries tend to be thinner, straighter, and gradually branching into smaller vessels, while pial veins are thicker, more curvy, and branching into vessels of all calibers^95^. Penetrating arterioles and venules were identified by tracking the pial vessels until they dive. This initial vessel identification was then further confirmed by pO_2_ measurements and OCT data (downward flow in arterioles and upward flow in venules). Capillaries were defined as microvessels a few branches away from penetrating vessels with diameter less than 10 µm.

### Estimation of CMRO_2_

For the mice in which we had both vascular pO_2_ and CBF measures over the same cortical region, we estimated the CMRO_2_ from CMRO_2_ = CBF([O_2_]_a_-[O_2_]_v_) (Fig. 6a). CBF was obtained from OCT data as total blood flow averaged through the depth of 50–100 µm. Vascular pO_2_ data from two-photon measurements was used to calculate the average arterial and venular O_2_ contents ([O_2_]_a_ and [O_2_]_v_) over the same depth using the equation [O_2_] = *α*.pO_2_ + *4 Hct*.*C*_*Hb*_.SO_2_^95^. *C*_*Hb*_ is hemoglobin content of RBC (5.3 µmole Hb/ml RBC)^95^ and *Hct* is hematocrit, assumed to be equal to the systemic hematocrit.

### O_2_ delivery by individual arterioles

In the mice with both vascular pO_2_ and Doppler OCT measures over the same region, we were able to detect arterioles for which we had both vascular pO_2_ versus depth and mean blood flow (Fig. 7a). A simple mass balance equation yielded O_2_ delivery from these arterioles to surrounding tissue (Fig. 7b):

2 $${\dot{O}}_{2}=-\,Q\frac{d[{O}_{2}]}{dz}$$

${\dot{O}}_{2}$ is O_2_ delivery to surrounding tissue per unit length of the vessel [µmol/min/µm], *Q* is mean blood flow in the arteriole, [O_2_] is oxygen content of the blood, and *z* is depth. Average dSO_2_/dz was obtained for each vessel over the depth of 0–150 µm and was converted to d[O_2_]/dz. Average flow in the vessel was also obtained over the same depth range.

### Systemic hematocrit

Systemic hematocrit was measured on a separate batch of age-matched mice (10 young, 9 middle-aged, and 8 old mice). Two hours after the beginning of the light cycle, blood was harvested from the saphenous vein of non-fasted mice and put in microhematocrit tubes by capillary action. Tubes were then sealed and spun in a microhematocrit centrifuge at high speed for 5 minutes. The length taken up by centrifuged red cells was measured and divided by the whole microhematocrit tube length to determine the hematocrit percentage.

### Statistical analysis

The results are presented as mean ± s.e.m. Statistical significance was calculated using ANOVA followed by Tukey HSD post hoc test. Statistical significance was assigned at *p < 0.05, **p < 0.01, ***p < 0.001, ****p < 0.0001 and *****p < 0.00001. The sample sizes were chosen empirically based on our previous experience.

### Code availability

Custom-written code in Matlab or Python are available from the corresponding author upon reasonable request.

### Data availability

The authors declare that the main part of data supporting the findings of this study is available within the paper and its supplementary figures. Data not presented within the paper or supplementary figures are available from the corresponding author upon reasonable request.

## Electronic supplementary material

Supplementary figures
