# Supplementary material for: Compromised microvascular oxygen delivery increases brain tissue vulnerability with age
Source: Sci Rep. 2018 May 29;8:8219. doi: 10.1038/s41598-018-26543-w (PMC5974237; doi:10.1038/s41598-018-26543-w)
Supplement: Supplementary file 1 — Supplementary figures [file 41598_2018_26543_MOESM1_ESM.pdf]

**Supplementary Information**

# Compromised microvascular oxygen delivery increases brain tissue vulnerability with age

Mohammad Moeini[1,2,3], Xuecong Lu[1,2], Pramod K Avti[1,2,7] , Rafat Damseh[1], Samuel Bélanger[1,2], Frédéric Picard[4], David Boas[5,6], Ashok Kakkar[3], Frédéric Lesage[1,2 *]

[1] Biomedical Engineering Institute, École Polytechnique de Montréal, Montréal, QC, Canada

[2] Research Center of Montreal Heart Institute, Montréal, QC, Canada

[3] Department of Chemistry, McGill University, Montréal, QC, Canada

[4] Centre de Recherche de l'Institut Universitaire de Cardiologie et Pneumologie de Québec (IUCPQ) , Québec, QC, Canada

[5] Athinoula A. Martinos Center for Biomedical Imaging, Massachusetts General Hospital, Harvard Medical School, Charlestown, MA, USA

[6] Biomedical Engineering Department, College of Engineering, Boston University, Boston, MA, USA

[7] Present address: Department of Biophysics, Postgraduate Institute of Medical Education and Research, Chandigarh, India

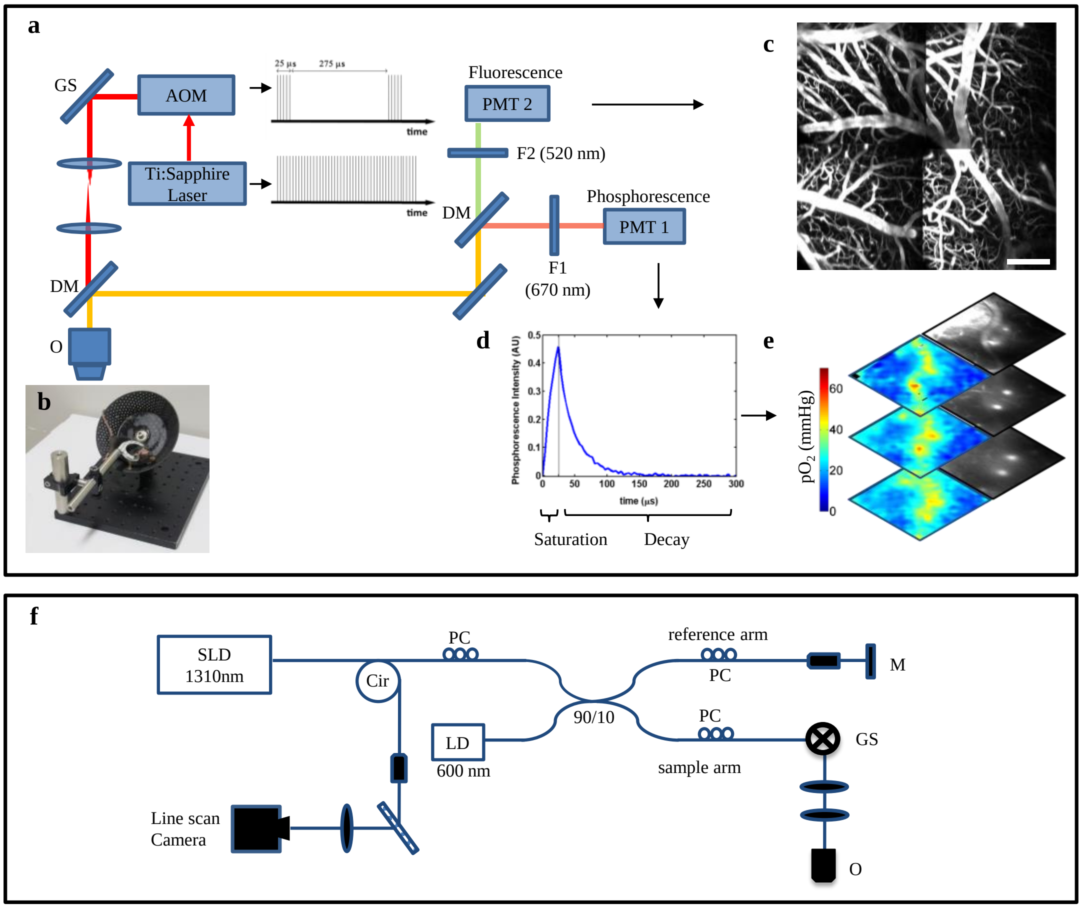


**Supplementary Figure 1.** Two-photon and OCT setups. **(a)** Simplified layout of the two-photon microscope. Laser beam was passed through an acousto-optic modulator (AOM) to adjust the gain and allow alternating "on" and "off" laser pulse periods. Two galvanometric scanners (GS) were used to scan the beam over x-y planes. Emitted light was collected by an objective (O). The phosphorescence and fluorescent photons were separated by dichroic mirrors (DMs), passed through filters (F1 and F2) and relayed to photomultiplier tubes (PMTs). **(b)** Imaging was performed under awake conditions using a custom-built treadmill wheel in which mice were allowed to walk or run freely while their head was restrained by a titanium bar. **(c)** Maximum intensity projection over 250μm thickness of four adjacent angiograms obtained by fluorescence imaging. Scale bar: 200 μm. **(d)** A representative averaged phosphorescence decay curve for a single point $pO_2$ measurement. **(e)** Examples of tissue $pO_2$ grid measurements over a 400μm× 400μm region at three depths obtained by phosphorescence lifetime microscopy, exhibiting clear tissue $pO_2$ gradient around penetrating arterioles. The color bar shows the $pO_2$ values in mmHg. **(f)** OCT setup: Light originating from a superluminescent diode (SLD) source was first sent to a circulator (Cir) and divided by a 10/90 fiber splitter into reference and sample arms. Scanning on the sample was performed using a dual galvanometer system (GS) combined with a telescope to be imaged using a 5x infinity corrected objective (O). In each arm, polarization control (PC) was integrated to maximize contrast. A custom built spectrometer based on a volume holographic grating was used as the detector with a high-speed line camera coupled to a 50 mm SWIR lens. A laser diode (LD) was also used for easy visualization of the exact scanning region.

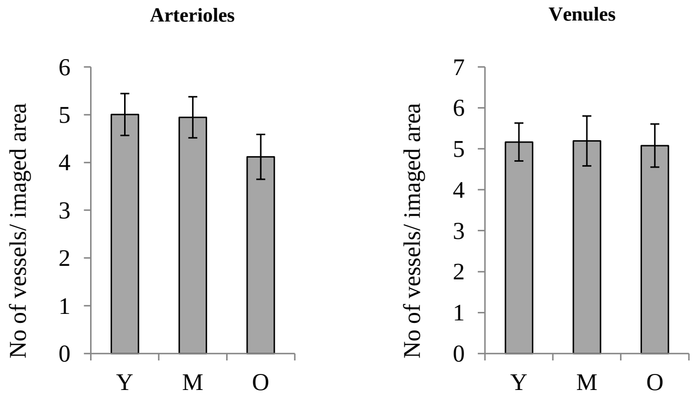


**Supplementary Figure 2.** Average (through the depth 50-650μm) number of detected arterioles (left) and venules (right) over the scanned region in Doppler OCT experiments. Results are presented as mean±s.e.m. No statistical significance (tested with ANOVA) was detected between the age groups. Y: young (n=14 mice); M: middle-aged (n=14 mice); O: old (n=15 mice).

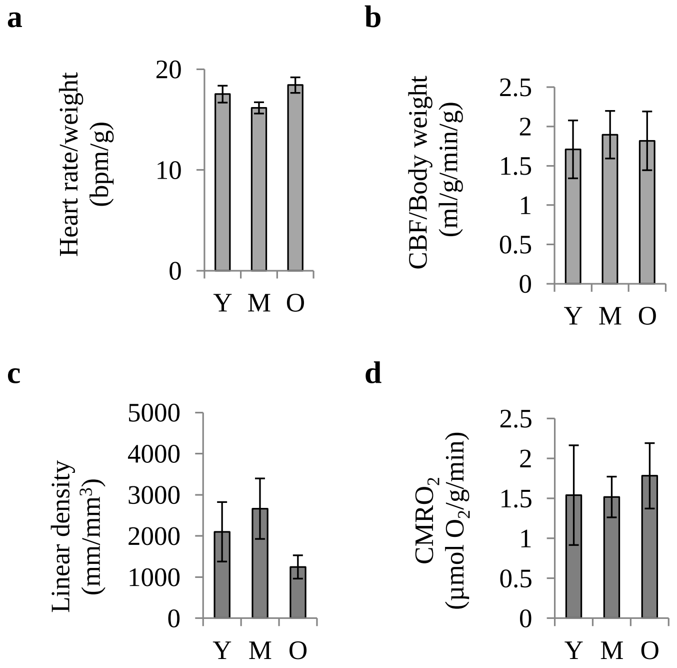


**Supplementary Figure 3.** Measured parameters normalized by total body mass. **(a)** Heart rate normalized by total body mass. **(b)** CBF normalized by total body mass. **(c)** Estimated capillary linear density from the simplified parallel capillary tubes model (Fig. 4j) using corrected CBF assuming that cortical thickness changes with age follow the body mass changes. **(d)** Estimated $CMRO_2$ from the vascular $pO_2$ and corrected CBF data, assuming that cortical thickness changes with age follow the body mass changes.
